# Oral *Porphyromonas gingivalis* and *Fusobacterium nucleatum* Abundance in Subjects in Primary and Secondary Cardiovascular Prevention, with or without Heterozygous Familial Hypercholesterolemia

**DOI:** 10.3390/biomedicines10092144

**Published:** 2022-08-31

**Authors:** Maria Cristina Curia, Pamela Pignatelli, Domenica Lucia D’Antonio, Damiano D’Ardes, Elena Olmastroni, Luca Scorpiglione, Francesco Cipollone, Alberico Luigi Catapano, Adriano Piattelli, Marco Bucci, Paolo Magni

**Affiliations:** 1 Department of Medical, Oral and Biotechnological Sciences, Università degli Studi “Gabriele d’Annunzio” di Chieti-Pescara, 66100 Chieti, Italy; 2 Department of Oral and Maxillofacial Sciences, “Sapienza” University of Rome, 00185 Rome, Italy; 3 Regional Center for the Study of Atherosclerosis, Hypertension and Dyslipidemia, “SS Annunziata” Hospital—ASL, 66100 Chieti, Italy; 4 C.A.S.T., Università degli Studi “Gabriele d’Annunzio” di Chieti-Pescara, 66100 Chieti, Italy; 5 Epidemiology and Preventive Pharmacology Service (SEFAP), Department of Pharmacological and Biomolecular Sciences, Università degli Studi di Milano, 20133 Milan, Italy; 6 IRCCS MultiMedica, Sesto S. Giovanni, 20099 Milan, Italy; 7 Master Course in Microsurgery in Odontostomatology, Saint Camillus International University for Health Sciences (Unicamillus), 00131 Rome, Italy; 8 Fondazione Villaserena per la Ricerca, 65013 Città Sant’Angelo, Pescara, Italy; 9 Casa di Cura Villa Serena, 65013 Città Sant’Angelo, Pescara, Italy

**Keywords:** atherosclerosis, *Porphyromonas gingivalis*, *Fusobacterium nucleatum*, cardiovascular disease, heterozygous familial hypercholesterolemia, secondary cardiovascular prevention

## Abstract

Background: Low-grade chronic inflammation, promoted by dysbiosis of the gut and oral microbiota, has been shown to contribute to individual susceptibility to atherosclerotic cardiovascular disease (ASCVD). High oral *Porphyromonas gingivalis* (*Pg*) and lower *Fusobacterium nucleatum (Fn)* concentrations have been associated with clinical and experimental atherosclerosis. We assessed oral *Pg* and *Fn* abundance in very high-risk patients with previously diagnosed ASCVD, with or without heterozygous familial hypercholesterolemia (HeFH), in subjects with HeFH in primary prevention and in healthy subjects. Methods: In this cross-sectional study, 40 patients with previously diagnosed ASCVD (10 with genetically proven HeFH, and 30 without FH), 26 subjects with HeFH in primary prevention, and 31 healthy subjects were selected to quantify oral *Pg* and *Fn* abundance by qPCR and assess oral health status. Results: Compared to healthy subjects, patients with previously diagnosed ASCVD showed greater *Pg* abundance (1101.3 vs. 192.4, *p* = 0.03), but similar *Fn* abundance. HeFH patients with ASCVD had an even greater *Pg* abundance than did non-HeFH patients and healthy subjects (1770.6 vs. 758.4 vs. 192.4, respectively; *p* = 0.048). No differences were found in the levels of *Pg* and *Fn* abundance in HeFH subjects in primary prevention, as compared to healthy subjects. Conclusions: Greater oral *Pg* abundance is present in very high-risk patients with previously diagnosed ASCVD, with or without FH, suggesting a potential relationship with CV events. Future studies will assess the predictive value of *Pg* abundance measurement in ASCVD risk stratification.

## 1. Introduction

Cardiovascular diseases (CVDs) are the leading cause of death in the world [1], and their known risk factors include diabetes, hypertension, smoking, and especially dyslipidemia and chronic low-grade inflammation. Among these factors, hypercholesterolemia, and specifically elevated low-density lipoprotein cholesterol (LDL-C), is an established causative factor for atherosclerotic CVD (ASCVD) [2]. The susceptibility to CVD reaches a high level of severity in patients with specific genotypes, which results in increased levels of LDL-C throughout their lives, such as familial hypercholesterolemia (FH). This is an autosomal dominant disease caused by mutations of gene encoding for low-density lipoprotein receptor (LDLR), apolipoprotein B (ApoB) or proprotein convertase subtilisin/kexin type 9 (PCSK9). The subjects carrying these mutations, even in heterozygosity (HeFH), present a much higher ASCVD risk due to the longstanding LDL-C burden [3,4]. Another high-risk cohort includes subjects with polygenic hypercholesterolemia, consisting of a series of mutations (single-nucleotide polymorphisms, SNPs) that individually have a lower impact, but which can increase, when added together, the circulating levels of LDL-C. The cumulative impact of these SNPs may be quantified by calculating an LDL-C SNP score [5]. Interestingly, the susceptibility to ASCVD differs among these patients, even when LDL-C levels are similar [6], suggesting that a pathogenetic role for ASCVD may be played by other factors. Among them, chronic low-grade inflammation, via the activation of the nucleotide-binding domain; the leucine-rich containing family, pyrin domain-containing-3 (NLRP3) inflammasome/interleukin 1-β (IL1-β) pathway [7,8] is a very important player, as demonstrated by the CANTOS study [9]. Chronic inflammation is promoted by different conditions, including dysbiosis of the gut and oral microbiota [10,11,12,13]. Evidence that alterations of the former are linked to dyslipidemia, inflammation. and ASCVD are available [14]. For the latter, some studies have shown the association of high concentrations of the odontopathogenic *Porphyromonas gingivalis* (*Pg*) and the commensal and periodontal pathogen *Fusobacterium nucleatum* (*Fn*) and periodontitis with clinical and experimental atherosclerosis [15]. Interestingly, greater subclinical carotid atherosclerosis has been reported to be associated with periodontitis in patients with systemic lupus erythematosus [16]. Moreover, periodontitis was found to be a significant risk factor for peripheral and carotid ASCVD in meta-analyses including large populations [17,18,19]. These observations suggest that pathological changes in oral microbiota composition and the related periodontitis may play a role in ASCVD pathophysiology by promoting chronic inflammation, dyslipidemia, including LDL-C oxidation [20], and the reduction of the antiatherogenic properties of high-density lipoprotein cholesterol (HDL-C) [21], endothelial-cell dysfunction [22], and, possibly, as-yet-unknown, additional pathological processes [23]. Notably, the overall periodontal condition of patients with periodontitis is mirrored by the relative abundance of total subgingival-plaque-specific bacteria in salivary microbiota [24], suggesting the reliability of sampling this latter biological matrix in this context.

To the best of our knowledge, no reports are available on the specific abundance of these bacterial strains in the oral microbiota of patients at high-risk for CVD in secondary CV prevention, with or without HeFH. Thus, in this study, we addressed the question of whether oral dysbiosis, namely, the altered abundance of *Pg* and *Fn*, was correlated with previous ASCVD (acute myocardial infarction (MI) and/or percutaneous coronary intervention (PCI)/drug-eluting stent (DES) or coronary artery bypass graft (CABG), carotid thromboendarterectomy (CEA)), in high-/very high-risk CVD patients, with or without HeFH.

## 2. Materials and Methods

### 2.1. Study Design and Population

This study had a cross-sectional design and included consecutive patients referred to the Regional Center for the Study of Atherosclerosis, Hypertension and Dyslipidemia (“SS Annunziata” Hospital—ASL Chieti, Italy; C.A.S.T., “G. D’Annunzio” University of Chieti, Italy) from September 2019 to October 2021. The study population included 40 patients (30 patients without HeFH and 10 patients with HeFH,) in secondary ASCVD prevention, as defined by previous MI with/without PCI-DES or double/triple CABG and/or CEA. All of these patients at high risk of CVD were optimally treated with an individual combination of hypocholesterolemic drugs (statins, ezetimibe, proprotein convertase subtilisin/kexin type 9 (PCSK9) inhibitors) at the maximally tolerated dose, according to the most recent guidelines [25], and anti-platelet drugs. When appropriate, other drugs, including anti-hypertensive, β-blockers, and others, were also used. Moreover, 26 sequential HeFH patients in primary CV prevention, treated with hypocholesterolemic drugs (statin, *n* = 2; statin+ezetimibe, *n* = 5; statin+PCSK9 inhibitor, *n* = 4; statin+ezetimibe+PCSK9 inhibitor, *n* = 8; ezetimibe+PCSK9 inhibitor, *n* = 1; no therapy, *n* = 5) were recruited. All HeFH patients received a genetic diagnosis related to their hypocholesterolemic condition. The genetic diagnosis highlighted the presence of causative mutations in heterozygosity, such as mutations on the LDLR, ApoB, or PCSK9 genes, typed according to Lipid inCode analysis results, within the LIPIGEN project [26]. We also enrolled 31 healthy subjects with normal circulating levels of TC and LDL-C (<200 mg/dL and <115 mg/dL, respectively) from recent history (the last 6–12 months) and/or at recruitment. All participants were subjected to a questionnaire about their lifestyle, evaluating alcohol consumption (g/day) and smoking habits (number of cigarettes/day). Their clinical histories have also been carefully assessed, specifically related to the presence of previous adverse cerebrovascular events (CVA), coronary artery disease (CAD) (myocardial infarction or angina), chronic renal failure (CKD), and hypertension (HTN), as well as concomitant medications. Inclusion criteria were as follow: patients in secondary CV prevention (previous MI or PCI) with genetic diagnoses of HeFH; subjects in primary CV prevention with genetic diagnoses of HeFH. Exclusion criteria were as follow: presence of chronic diseases known or detected at screening (except ASCVD), with known gastro-intestinal diseases also related to intestinal dysbiosis. Additionally, we excluded subjects who had undergone antibiotic therapy in the previous 3 months, who were pregnant or breastfeeding, who had systemic pathologies associated with immunological impairment (type 1 diabetes mellitus, organ transplants, or concomitant malignancies), the intake of immunosuppressive drugs, and previous chemotherapy and/or radiotherapy.

The study was performed in accordance with the guidelines of the Declaration of Helsinki. The study was approved by the Ethics Committee of the Provinces of Chieti and Pescara (Italy) (Approval n. 4, dated 25 February 2016). Written, informed consent was obtained from each subject.

### 2.2. Clinical Procedures

Clinical procedures were performed for all study subjects, who underwent a fasting venous blood sampling and a full clinical examination, including the determination of height, body weight, body mass index (BMI), waist circumference (by means of a non-stretchable tape at the umbilical level (standing position), heart rate, and arterial blood pressure. A further investigation was made to assess the oral health of all participants (see below).

### 2.3. Oral Examination

Oral examination of patients and healthy subjects was performed by a trained dentist (P.P.) using mirrors (MIR3HD, Hu-Friedy, Chicago, IL, USA), a dental probe (PCP-UNC 15, Hu-Friedy, Chicago, IL, USA), and an intra-oral light. Oral health status was assessed by indices recorded on the standardized Oral Health Questionnaire: number of teeth, plaque index (PI), and gingival index (GI). PI and GI were measured on six surfaces (buccal–mesial, mid-buccal, buccal–distal, lingual–mesial, mid-lingual, and lingual–distal) on the Ramfjord teeth (the maxillary right first molar, the maxillary left central incisor, the maxillary left first premolar, the mandibular left first molar, the mandibular right central incisor, and the mandibular right first premolar) with a PCP-UNC 15 probe [27]. The presence of mobile and/or fixed prostheses was recorded with an index from 2 to 0 respectively: 0, absence of prosthesis; 1, single implant/crown or only upper/lower mobile prosthesis; 2, multiple implants/crowns or a double mobile prosthesis.

### 2.4. Collection of Oral Samples

Oral samples were collected in the morning, 12 h after the last tooth-brushing and 2 h after the last food/liquid intake. All patients and healthy subjects underwent brushing of the middle third dorsum of the tongue by the same operator (dentist, P.P.) under the same conditions. Lingual brushing was performed using a sterile microbrush (Cura Farma, Italy) and was repeated 5 times, following the posterior–anterior direction, reaching 2/3 of the tongue. After sampling, the microbrush was shaken for 30 s, immediately placed in a tube containing 5.0 mL of phosphate-buffered saline (PBS), and then stored at 4 °C until nucleic acid extraction was performed.

### 2.5. Clinical Biochemistry

After blood sampling, plasma samples were immediately separated by centrifugation, and aliquots were immediately stored at − 20 °C for subsequent assays. In each plasma sample, TC, TG, HDL-C, apoAI, apoB, Lp(a), fasting plasma glucose (FPG), uric acid, aspartate aminotransferase (AST), alanine aminotransferase (ALT), gamma-glutamyltranspeptidase (GGT), and creatine phosphokinase (CPK) isoenzymes were measured according to standard automated clinical procedure. LDL-C was calculated according to the Friedewald formula. Non-HDL-C was calculated as TC minus HDL-C.

### 2.6. DNA Isolation and Analysis of Oral Bacterial Strains

After the collection of the oral biological samples from patients and healthy subjects, molecular analyses were performed to quantify the following bacterial strains: *Porphyromonas gingivalis* (*Pg*) ATCC 33277 and *Fusobacterium nucleatum* (*Fn*) ATCC 25586 (LGC Standards S.r.l., Sesto San Giovanni, Milano, Italy) so as to assess the presence of any imbalances in the oral microbial flora. The total genomic DNA from the samples and from the bacterial strains were extracted, as previously reported [28]. Molecular analyses to quantify *Pg* and *Fn* abundances in oral samples was performed by qPCR, as previously reported [29].

### 2.7. Statistical Analysis

Continuous variables are presented as medians and interquartile ranges (Q1 and Q3), whereas categorical variables are presented as percentage rates (%). Differences in median values between groups were assessed by a Wilcoxon Rank-Sum test, while a chi-squared test was used to determine independency among categorical variables. Correlations between *Pg* abundance and *Fn* abundance with risk factors were assessed using Spearman’s rank coefficient, and the Loess procedure was used to fit a smooth curve to the data, which attempts to capture the general pattern. All tests were 2-sided, and *p*-values < 0.05 were considered statistically significant. All analyses were performed using Statistical Analysis System software, version 9.4 (Statistical Analysis System Institute, Inc, Cary, NC, USA) and R Software, version 4.1.2 (The R Foundation for Statistical Computing c/o Institute for Statistics and Mathematics, 1020 Vienna, Austria). Data retrieval, analysis, and manuscript preparation were solely the responsibility of the authors.

## 3. Results

### 3.1. General Characteristics of the Study Cohort

The baseline data of the study cohort are reported in Table 1. The group of patients with previous MI or PCI included both HeFH and non-FH subjects, who were significantly older than healthy subjects (*p* = 0.001) and were mostly males (females: 22.5%; *p* = 0.011). They also showed a greater prevalence of being overweight or obese (*p* = 0.012), compared to healthy subjects. Patients in secondary CVD prevention did not differ from healthy subjects for PI, GI, number of teeth, or presence of prostheses. The healthy subject group included more smokers (19% vs. 5%, *p* = 0.058) (Table 1).

### 3.2. Analysis of Pg and Fn Abundance: Correlation with CVD Status

In the whole cohort, *Pg* abundance was significantly greater in patients with previously diagnosed CVD (*p* = 0.03) than in healthy subjects, while *Fn* abundance and *Fn*/*Pg* ratio did not differ between groups (Table 2A). After stratification of the secondary CVD prevention cohort according to the presence (or absence) of HeFH, we observed a stepwise increase in *Pg* abundance among classes, with the subgroup of HeFH patients showing the greatest *Pg* abundance (*p* = 0.048) (Table 2B).

Since age, sex, and BMI significantly differed between the healthy subjects group and the CVD patients group (Table 1), we further stratified each group according to these variables. Stratification by age was conducted by taking the age of 60 years, which is the median age of the whole cohort (60 years (54–67)), as the cut-off value. As shown in Table 3, although the *Pg* abundance median was always greater in CVD patients, whether or not a subject was above 60 years of age, compared to healthy subjects, such a difference did not reach statistical significance. The same situation was observed for *Fn* abundance and *Fn*/*Pg* ratio. Only in those below 60 years of age was the plaque index found to be significantly higher (*p* = 0.02) in CVD patients than in healthy subjects.

Stratification of the two groups by sex did not allow us to highlight significant differences between healthy controls and CVD patients (Table 4), suggesting that sex is not a confounding factor.

Stratification of the two groups by a BMI less than or equal to/greater than 30 kg/m^2^ showed that *Pg* abundance was significantly (*p* < 0.03) greater in CVD patients with BMIs below this threshold, and thus either of a normal weight or overweight, but not with obesity (Table 5). Such a difference was lost when a BMI was above 30 kg/m^2^, which could also possibly be due to the small number of subjects in these subgroups.

**Figure 1:**
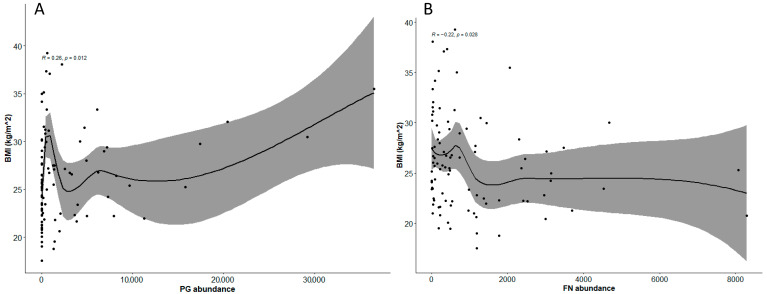
Correlation between BMI and *Pg* abundance (panel **A**) and *Fn* abundance (panel **B**) in the whole study cohort.

Based on our results, BMI seems to be a confounding factor for *Pg* abundance distribution. Thus, we further investigated the correlation between *Pg* abundance and BMI levels using Spearman’s rank coefficient analysis. In the whole study cohort (including healthy subjects, HeFH subjects in primary CVD prevention, and non-FH patients in secondary CVD prevention), BMI was directly correlated with *Pg* abundance (R = 0.26, *p* = 0.012) (Figure 1A). In contrast, BMI was negatively correlated with *Fn* abundance (R = −0.22, *p* = 0.028) (Figure 1B).

In addition, *Pg* abundance remained significantly greater in the whole CVD patient group when smokers in both the healthy subjects and patient groups were excluded (*p* = 0.03) (Table 6).

A group of HeFH patients in primary prevention (*n* = 26; age: 53.5 (32–55) years; females: 46%; BMI: 25.2 (22.32–29.06) Kg/m^2^; smokers: 15.4% (Median (IQR)), followed at the Lipid Clinic for >5 years, were also taken into consideration. Their *Pg* and *Fn* abundances was comparable to those of healthy subjects (*Pg*: 10.5 (0–413.2) vs. 192.4 (0–1364.6), *p* = 0.41; *Fn*: 492.9 (280.4–1189.2) vs. 324.2 (34.9–1438.8), *p* = 0.35, respectively) (Table 7). The *Fn*/*Pg* ratio was significantly higher in the HeFH group.

## 4. Discussion

ASCVD is the result of several concomitant causative factors that are individually associated. In addition to elevated circulating LDL-C levels, whether due to lifestyle factors, polygenic conditions, or monogenic disease, such as HeFH, the pathophysiology of ASCVD also includes chronic low-grade inflammation generated by different components, including oral and gut microbiota changes [30].

In the present study, we explored the abundance of the periodontal pathogenic bacteria *Pg* and *Fn* in patients with an established pathological phenotype: previous ASCVD (MI or PCI), with or without HeFH. Patients with ASCVD tended to have more fixed prostheses (crowns and implants) than healthy subjects; this condition indicates previous tooth loss, infections, and caries. The main results suggest that these patients with ASCVD showed a significantly greater *Pg* abundance, compared to healthy subjects and HeFH subjects in primary prevention, independently of age and sex. Interestingly, having a BMI lower than 30 kg/m^2^ was associated with greater *Pg* abundance, possibly indicating a major contribution of related chronic inflammation to ASCVD, while such pro-inflammatory patterns in subjects with obesity may derive from a more complex scenario that may also include the role of dysfunctional adipose tissue and the related adipokines and pro-inflammatory cytokines [31,32]. In the whole study cohort, BMI, another established ASCVD risk factor, was correlated positively with *Pg* abundance, in agreement with previous reports [33,34], and it was correlated negatively with *Fn* abundance.

Periodontitis is one of the most prevalent forms of chronic inflammation of the oral cavity and has been reported to be associated with systemic diseases. Specifically, periodontal disease has been linked to an increase in the risk of ASCVD incidence [35]. Periodontal pathogenic bacteria, such as *Pg* and *Fn*, can accelerate atheroma formation directly, by entering the circulation and accelerating coronary or aortic atherosclerosis [36,37,38], or indirectly, by disrupted lipoprotein profiles and lipid deposition [30]. In patients with periodontitis, increased levels of proinflammatory lipids and decreased levels of anti-inflammatory lipids were observed [39]. Importantly, periodontitis could increase ASCVD risk, especially in high-risk subjects, such as patients with HeFH.

In the present study, patients with previously diagnosed ASCVD showed a significantly greater *Pg* abundance, but no differences in *Fn* abundance were noted, as compared with healthy subjects and HeFH subjects in primary prevention. Interestingly, the observed difference was still present after exclusion of the (few) smokers from both groups. We enrolled consecutive patients; the median age resulted as statistically higher in CVD patients than in healthy subjects. Periodontal disease affects more than 50% of the United States’ population over the age of 30 years, and the anaerobic bacterium *Pg* is known to be a major player in inducing dysbiosis of the oral microbiome [40]. Given that the median age of both groups, although significantly different, spanned well above the age of 30, we may consider such an age difference as being of no clinical relevance, as shown by comparison analysis. Few data are available regarding the occurrence of sex differences as related to oral odontopathogenic bacteria. While in our study sex did not appear to influence *Pg* or *Fn* abundance in any group, it has recently been reported that, in a cohort of young people aged 32 years, a greater proportion of *Pg* at subgingival sites was present in females as compared to males [41].

Interestingly, in the HeFH subgroup in secondary prevention, a *Pg* abundance even greater than that of non-HeFH patients with ASCVD was observed. Although it is not possible to causally correlate the currently assessed greater *Pg* abundance with ASCVD occurrence, one may speculate that the concomitant presence of abundant odontopathogenic strains, such as *Pg*, if present for years, may have contributed to a further increase in ASCVD risk in subjects with HeFH, which is, per se, a very high-risk, lifelong condition. Supporting this hypothesis, in the HeFH group in primary prevention, no differences in *Pg* and *Fn* abundance were found as compared with healthy subjects.

Interestingly, in the whole study cohort, BMI, another well-established ASCVD risk factor, was correlated positively with *Pg* abundance and negatively with *Fn* abundance. It has been previously reported that the number of Red Complex oral bacteria, including *Pg*, was higher in subjects with high BMIs or waist circumferences, independent of periodontitis [30]. A bidirectional relationship between obesity and periodontal disease has also been proposed. Oral bacteria may alter the endocrine functions of brown adipose tissue [42], exacerbate inflammation due to obesity [43], induce weight gain, and increase adipose tissue in diet-induced obesity in mice [44] through endotoxemia and the activation of immune cells. Local inflammation, such as periodontitis, could affect energy regulation, and equally, obesity could exacerbate periodontal disease [45]. Conversely, the amount of *Fn* was not associated with previous ASCVD and showed an inverse relationship with BMI, thus suggesting that it may not be included among the risk factors for CVD. Any (negative) association of *Fn* with BMI has not yet been reported, indicating that our observation is novel. Of note, higher proportions of several periodontal pathogens, including *Fusobacterium nucleatum ss vincentii*, have been reported in obese patients with chronic periodontitis [46]. Oral *Pg* infection may have played a role in the pathogenesis of CVD by increasing systemic inflammation and deteriorating lipid metabolism, particularly in the context of high-risk patients or HeFH. A similar increase of serum LDL-C would alter the cholesterol component in the cell membrane and cytoplasm of macrophages, which could further affect recognition of *Pg* by immune cells. Increased production of tumor necrosis-α, interleukin-6, monocyte chemoattractant protein-1, and inducible nitric oxide synthase may also facilitate colonization and proliferation of *Pg* in the oral niche in the hyperlipidemic host [47].

Supporting our data, a relationship between MI and an increase in *Pg-*targeting antibodies in secondary prevention patients with periodontitis has also been proposed [48]. Conversely, *Fn* has a reported role in cancer diseases, but it does not appear to have an association with ASCVD [28,49]. In any case, the role of *Fn* in atherosclerosis and CVD still remains controversial [36,50] and needs further clinical study.

According to the findings of this study, it remains to be established as to whether the increase in *Pg* abundance observed in ASCVD patients, but not in healthy subjects or patients with HeFH in primary prevention, is likely to be a consequence (almost a “marker”) rather than a cause of ASCVD. Alternatively, this increase in *Pg* could gradually manifest itself with the deterioration of cardiovascular homeostasis. After all, today we are still wondering how and when *Pg* intervenes and whether it is actually causative of CVD, or not. Perhaps *Pg* could have a pathogenic role, possibly accelerating (more than causing) a pathological process that has already been activated, as proposed by Maekawa et al. (“periodontal infection would determine the acceleration of the atherosclerotic process, rather than its genesis, in metabolically susceptible organisms such as ApoE-/-mice” [32]). Therefore, there could be a balance between *Pg* and *Fn* abundance: the more labile it is, the worse the oral and cardiovascular health of the subject is. *Pg* is present at the highest level in HeFH patients—those who are already predisposed to risk, and those who have actually had a CVD event (secondary prevention). Most recruited patients, both in primary and secondary prevention, were on statin treatment, which has been shown to be associated with a lower prevalence of gut microbiota dysbiosis [51]. The question then arises as to whether such treatment may somehow also affect oral microbiota composition.

A limitation of this study is its real-world nature with the recruitment of consecutive ASCVD patients, who did not fully match the healthy controls according to age and sex. These differences have been addressed above. Moreover, the results of the microbiota analysis cannot be retrospectively extended to the previous years of life of the participants, and therefore, any causal relationship between oral microbiome composition and ASCVD cannot be established and may only be hypothesized. According to this study, however, a practical suggestion may be that some oral microbiota analyses might be useful for the accurate initial CVD risk-stratification of specific high-risk subjects, such as those with HeFH. Another limitation of the study, due to the complex multidisciplinary activities associated with the study design, is the relatively limited size of the study cohort, preventing us, for example, from highlighting potential sex differences. Moreover, due to the complex drug treatment regimens of all patients with ASCVD, any potential association between biochemical markers, including lipid markers, and *Pg* and *Fn* abundance could not be explored. In this study, we explored the abundance of two components of the oral microbiome, *Pg* and *Fn*. Although they are recognized as very relevant in the association with atherosclerosis development, the mapping of a large set of bacterial components of the oral microbiome may provide further insights

In conclusion, taking this study together with the available evidence, the role of *Pg* as related to the risk of CV events appears to be relevant, while the role of *Fn* appears to be complex and multifaceted. Based on these observations, as a future perspective, patients in primary prevention could be monitored over time to assess whether any adverse event is preceded by an increase in oral *Pg* concentration. As a general statement, it is therefore advisable to also implement CVD prevention through periodontal prophylaxis, intensive periodontal therapy, and therapeutic interventions.

## Figures and Tables

**Table 1:** General features of healthy subjects and CVD patients.

	HEALTHY SUBJECTS	CVD PATIENTS +	*p*-Value
n	31	40	
Age * (years)	57 (52–64)	66 (60–69)	0.001
Female (%)	51.61	22.5	0.01
BMI * (kg/m^2^)	23.53 (21.60–27.10)	26.6 (24.31–29.99)	0.01
Type 2 diabetes mellitus/IGT (n/n)	0/0	15/10	
Smoking (%)	19.4	5	0.06
Plaque index *	1 (0.75–1.58)	1.28 (1.0–1.4)	0.15
Gingival Index *	0.75 (0–1)	0.5 (0.12–0.775)	0.64
Number of Teeth *	25 (23–26)	22.5 (16.5–26)	0.17
Removable denture (%) **			0.28
0	83.87	67.5	
1	12.9	27.5	
2	3.23	5	0.07
Fixed prosthesis (%)	35.48	57.5	0.07

IGT: impaired glucose tolerance, * Median (IQR), ** Removable denture: 0, absence of prosthesis; 1, single implant/crown or only upper/lower mobile prosthesis; 2, multiple implants/crowns or a double mobile prosthesis. + CVD patient group included 10 patients with heterozygous familial hypercholesterolemia (HeFH) and 30 patients without HeFH.

**Table 2:** *Pg* and *Fn* abundance: Comparison between healthy subjects and (**A**) all patients with previously diagnosed CVD, and (**B**) with the non-HeFH subgroup and the HeFH subgroup.

A.	HEALTHY SUBJECTS (*n* = 31)	CVD PATIENTS (All) (*n* = 40)		*p*-Value
*Pg* abundance *	192.38 (0–1364.57)	1101.25 (20.18–4945.35)		0.03
*Fn* abundance *	324.15 (34.85–1438.78)	364.45 (78.91–1368.23)		0.70
Fn/Pg *	0.22 (0.04–1.57)	0.37 (0.04–1.79)		0.66
**B.**	**HEALTHY** **SUBJECTS** **(*n* = 31)**	**CVD PATIENTS** **(excl. HeFH) (*n* = 30)**	**CVD PATIENTS** **(HeFH Only) (*n* = 10)**	***p*-Value**
*Pg* abundance *	192.38 (0–1364.57)	758.35 (0.81–4938.69)	1770.58 (531.75–4952)	0.048
*Fn* abundance *	324.15 (34.85–1438.78)	364.45 (81.04–736.03)	360.36 (52.17–3130.65)	0.78
*Fn*/Pg *	0.22 (0.04–1.57)	0.42 (0.04–3.4)	0.34 (0.06–0.51)	0.83

* Median (IQR).

**Table 3:** *Pg* and *Fn* abundance: Comparison between healthy subjects and all patients with previously diagnosed CVD according to an age of less than (**A**) or equal to/greater than (**B**) 60 years.

A.	HEALTHY SUBJECTS (*n* = 21)	CVD PATIENTS (All) (*n* = 12)	*p*-Value
*Pg* abundance *	2.90 (0–376.58)	411.46 (0–2917.45)	0.31
*Fn* abundance *	734.25 (146.37–1772.17)	464.96 (116.12–1564.81)	0.84
*Fn*/Pg *	0.60 (0.15–2.58)	0.49 (0.33–0.93)	0.99
Plaque index	1 (0.66–1.42)	1.58 (1.1–2.04)	0.02
**B.**	**HEALTHY SUBJECTS** **(*n* = 10)**	**CVD PATIENTS (All)** **(*n* = 28)**	***p*-Value**
*Pg* abundance *	1016.98 (192.38–3813.77)	1449.84 (225.96–5514.5)	0.73
*Fn* abundance *	131.38 (26.83–447.14)	289.84 (78.91–1368.23)	0.19
*Fn*/Pg *	0.05 (0.01–0.27)	0.13 (0.04–2.81)	0.16
Plaque index	1.29 (0.75–1.92)	1.25 (0.9–1.6)	0.86

* Median (IQR).

**Table 4:** *Pg* and *Fn* abundance: Comparison between healthy subjects and all patients with previously diagnosed CVD according to sex ((**A**), males, and (**B**) females).

A.	HEALTHY SUBJECTS (*n* = 15)	CVD PATIENTS (All) (*n* = 31)	*p*-Value
*Pg* abundance *	93.24 (0–1364.57)	813.79 (19.1–6858.2)	0.08
*Fn* abundance *	181.92 (23.74–527.69)	384.95 (91.35–2356.73)	0.10
*Fn*/Pg *	0.24 (0.13–0.6)	0.43 (0.06–1.79)	0.88
Plaque index	1.33 (0.66–1.67)	1.39 (1.08–1.75)	0.34
**B.**	**HEALTHY SUBJECTS** **(*n* = 16)**	**CVD PATIENTS (All)** **(*n* = 9)**	***p*-Value**
*Pg* abundance *	366.22 (0–1762.26)	1462.91 (60.9–3082.05)	0.28
*Fn* abundance *	941.78 (37.32–2703.18)	208.5 (52.17–467.54)	0.24
*Fn*/Pg *	0.14 (0.03–2.58)	0.35 (0.03–4.09)	0.90
Plaque index	1 (0.79–1.5)	1 (1–1.11)	0.76

* Median (IQR).

**Table 5:** *Pg* and *Fn* abundance: Comparison between healthy subjects and all patients with previously diagnosed CVD according to BMI less than (**A**) or equal to/greater than (**B**) 30 kg/m^2^.

A.	HEALTHY SUBJECTS (*n* = 25)	CVD PATIENTS (All) (*n* = 30)	*p*-Value
*Pg* abundance *	27.88 (0–1322.5)	1360.39 (19.21–4952)	0.03
*Fn* abundance *	527.69 (146.37–1438.78)	377.94 (81.04–1781.42)	0.98
*Fn*/Pg *	0.42 (0.13–2.58)	0.39 (0.05–3.4)	0.75
Plaque index	1 (0.66–1.42)	1.25 (1–1.6)	0.08
**B.**	**HEALTHY SUBJECTS** **(*n* = 4)**	**CVD PATIENTS (All)** **(*n* = 10)**	***p*-Value**
*Pg* abundance *	1495.12 (582.4–11319.21)	741 (223.82–4680.61)	0.73
*Fn* abundance *	30.84 (19.13–37.32)	266.61 (30.97–667.8)	0.10
*Fn*/Pg *	0.02 (0.01–0.04)	0.37 (0.04–1.02)	0.06
Plaque index	1.29 (0.96–1.75)	1.35 (1.03–1.75)	0.94

* Median (IQR).

**Table 6:** *Pg* and *Fn* abundance: Comparison between healthy subjects and all patients with previously diagnosed CVD, after the exclusion of smokers.

	HEALTHY SUBJECTS (*n* = 25)	CVD PATIENTS (All) (*n* = 38)	*p*-Value
*Pg* abundance *	27.88 (0–1322.5)	1101.25 (21.15–4952)	0.03
*Fn* abundance *	324.15 (34.85–1194.65)	364.45 (81.04–1446.59)	0.56
*Fn*/Pg *	0.23 (0.04–1.57)	0.37 (0.04–1.79)	0.72
Plaque index *	1.16 (0.75–1.67)	1.28 (1–1.67)	0.32

* Median (IQR).

**Table 7:** *Pg* and *Fn* abundance: Comparison between healthy subjects and HeFH patients in primary prevention.

	HEALTHY SUBJECTS (*n* = 31)	HeFH PATIENTS IN PRIMARY PREVENTION (*n* = 26)	*p*-Value
*Pg* abundance *	192.38 (0–1364.57)	10.45 (0–413.2)	0.41
*Fn* abundance *	324.15 (34.85–1438.78)	492.94 (280.41–1189.15)	0.35
*Fn*/Pg *	0.22 (0.04–1.57)	3.68 (0.23–37.92)	0.03

* Median (IQR).

## Data Availability

Data will be available upon request.
